# Correlation of Foot Bimalleolar Angle and Ultrasonography in Assessing the Severity of Club Foot in Neonates Treated by the Ponseti Method

**DOI:** 10.5704/MOJ.1811.003

**Published:** 2018-11

**Authors:** V Bajaj, R Anshuman, N Verma, MP Singh, A Tandon

**Affiliations:** Department of Orthopaedics, University College of Medical Sciences, Delhi, India

**Keywords:** CTEV, ultrasound, foot bimalleolar angle, Pirani score, Ponseti method

## Abstract

**Introduction:** Correlation of Pirani score and foot bimalleolar (FBM) angle has been used in few studies but correlation of FBM angle with ultrasonography has never been evaluated so they are being correlated in assessing the severity of clubfoot in neonates treated by Ponseti method.

**Material and Methods:** Thirty-two feet with congenital talipes equinovarus (CTEV) deformity in neonates were prospectively treated by the Ponseti method. FBM angle and ultrasound parameters were measured three times i.e. at the time of initial presentation, at four weeks of treatment and at completion of treatment. The feet were divided according to the Pirani score in groups: one (0-2.0), two (2.5-4) and three (4.5-6). Correlation between FBM angle and ultrasound parameters were evaluated using Pearson correlation/regression.

**Results:** Correlation between FBM angle and ultrasound parameters were statistically significant (p-value < 0.05).

**Conclusion:** Ultrasound has the potential to accurately depict the pathoanatomy in clubfoot. FBM angle and ultrasound are objective methods to assess the severity of clubfoot. FBM angle and ultrasonography correlated in severity of deformity and correction achieved along the course of treatment.

## Introduction

Idiopathic clubfoot, also known as “Congenital Talipes Equinovarus” or “CTEV” is one of the commonest congenital foot anomalies. The incidence of CTEV varies from 0.9/1000 to 4-7/1000 live births with bilateral cases accounting for 50% of the cases. There exist many modalities of treatment for this entity that includes correction by serial plaster casts as described by Kite and Ponseti and various surgical procedures with satisfactory results^1-5^. Ponseti method of serial manipulation by weekly cast is currently the gold standard for treatment of clubfoot in infants, with excellent results^6^.

The myriad questions by apprehensive parents regarding the extent of deformity, progress of correction, outcome and need for surgery necessitates the need for objective evaluation of the disorder. The clinical evaluation of clubfoot assessed by various scores like (1) Pirani score (PS) (2) Dimeglio score (3) Catterall score (4) Harold and Walker score are subjective in nature, have interobserver and intraobserver variations and they do not give objective evidence of the severity of deformity^7^. It is seen that in about 15% of cases spurious corrections can occur in which clinical correction is seen but underlying anatomy is still disturbed which may be the cause of recurrence^8-9^. Thus, there is a need for an objective evaluation of clubfoot correction during Ponseti treatment.

Foot bimalleolar angle (FBM) is a combined indicator of forefoot adduction and hind foot varus. The anteromedial angle between the long axis of foot and the bimalleolar plane is taken as FBM angle. It is an objective criterion to measure the severity of clubfoot. Its normal value is 82.5 degrees in infants^10^. Serial measurements can be used to assess the severity of deformity and correction achieved.

Various studies have analysed the role of radiography for assessing the correction achieved but have found the angles not correlating with deformity. This may be because of non-visualisation of un-ossified tarsal bones. Sonography is an alternative imaging modality for assessing severity of clubfoot. It offers advantage over radiography in the assessment of un-ossified cartilaginous structures, bones and surrounding soft tissues. This provides a reliable objective method to assess the deformity and to identify spurious corrections if any^6^.

Correlation of Pirani score and FBM angle has been used in a few studies ^11, 12^ but correlation of FBM angle with ultrasonography has not been evaluated during treatment of CTEV by Ponseti method, hence the aim of this study.

## Materials and Methods

Thirty-two feet of 30 children with CTEV deformity were prospectively treated by the Ponseti method from June 2013 to April 2015 on outpatient basis in the CTEV clinic at a tertiary care centre, after obtaining clearance from institutional ethical committee and consent of the parents/guardian of the patients and were included in this prospective level IV study. All cases of previously/partially treated and/or secondary clubfoot (associated other congenital abnormalities like spina bifida, arthrogryposis multiplex congenital, cerebral palsy) as well as those whose parents or guardians did not give consent were excluded. The feet were graded clinically by the Pirani score which is a simple scoring system based on six clinical signs^13^.

**Fig. 1:**
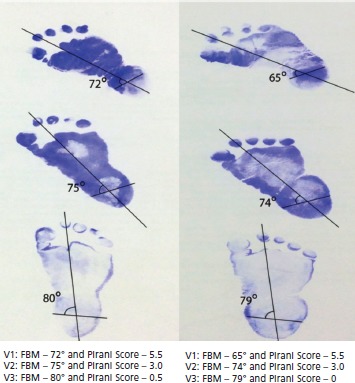
Foot bimalleolar angle (FBM) at initial visit, second visit and final visit along the course of treatment.

For FBM angle, keeping the foot in weight bearing position, foot tracings were taken on a plain white paper. Simultaneously the mid-points of both malleoli were marked on the same foot print by placing a pencil on both sides. Long axis was drawn taking second toe and midpoint of most broad part of heel as two reference points. Bimalleolar axis, the line joining both the malleoli, were drawn and where it intersected with the long axis of foot forming an angle was defined as the FBM angle (Fig. 1). Podograms were made by placing the plantar surface of the feet on inkpad and then firmly placing them on a clean sheet of paper to have good contact of heel, lateral border of foot and all the toes. Any imprints not having all three delineated clearly were discarded to ensure uniformity.

*Podograms showing calculation of FBM

**Fig. 2:**
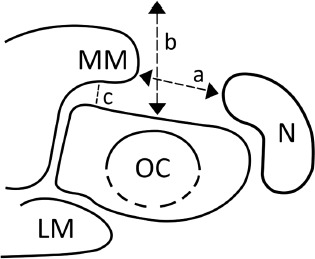
Osteocartilaginous relationship on medial projection.

The involved foot of the child was subjected to ultrasound by an experienced radiologist and following parameters were documented. On the medial projection the transducer was kept more vertical on the medial border of foot in the line of tibia to match the equinus deformity and the following parameters were calculated - medial malleolus navicular distance (MMN) and medial soft tissue thickness (MST) (Fig. 2). On the dorsal projection the transducer was kept at the dorsal aspects in various positions of foot, and the length of tendo-achilles (TAL) was calculated. On the lateral projection the transducer was kept on the lateral border of foot, and the calcaneocuboid distance (CCD) and calcaneocuboid angle (CCA) were calculated.

MM=medial malleolus; LM=lateral malleolus; N=navicular; OC= ossification centre of talus; a=distance between the medial malleolus and the navicular (MMN distance); b=medial soft tissue thickness; c=soft tissue thickness within the ankle mortise.

Corrective serial casting by the Ponseti method was applied and the child was followed up at weekly intervals for re-manipulation and change of cast till full correction was achieved. Pirani score, ultrasonography and FBM angle were evaluated in the beginning of treatment, at four weeks and at full correction of deformity with or without tenotomy, irrespective of time duration. Correlation between FBM angle and ultrasound parameters was determined using Pearson correlation/regression test.

## Results

This study included treatment of 30 children; two cases were bilateral and 28 were unilateral (total: 32 feet) who were managed by the Ponseti method. Pirani score, FBM angle and ultrasound parameters were measured three times, that is at the time of initial presentation, at four weeks of treatment and at completion of treatment.

The mean of FBM angles recorded at baseline was 66.55 (SD, 2.06), at four weeks was 70.83 (SD, 3.15) and at completion of treatment was 79.23 (SD, 1.36). It was found that FBM angle went on increasing with serial treatment and approached close to normal (82.5 degrees). In sonographic evaluation both the ossified and non-ossified structures were well visualised. The mineralised part of talar bones appeared as highly reflective surface with acoustic shadowing. The non-ossified cartilaginous structures were poorly echogenic with evenly spaced bright foci within. The articular cartilage appeared totally anechoic. Using all the three projections various parameters were measured all the three times of ultrasonic evaluation.

Using Pearson correlation it was found that FBM angle showed positive correlation with medial malleolus navicular (MMN) distance and length of achilles tendon (TA) i.e., these parameters increased as FBM angle increased with serial casting along the course of treatment. Correlation was significant (p-value <0.01) (Table I).

Using Pearson correlation it was found that the FBM angle showed negative correlation with medial soft tissue thickness (MST), calcaneocuboid angle (CCA) and calcaneocuboid distance (CCD) that is, these parameters decreased as FBM angle increased with serial casting along the course of treatment. Correlation was significant (p-value <0.01) (Table I).

**Table I:** The correlation of foot bimalleolar (FBM) angle with various sonographic parameters and among each other (p-values)

Parameters	FBM	MMN	MST	CCA	CCD	TA
FBM angle (degree)	1	0.75	-0.60	-0.76	-0.68	0.75
MMN distance (mm)	0.75	1	-0.44	-0.76	-0.58	0.74
MST (mm)	-0.60	-0.44	1	0.70	0.65	-0.41
CCA (degree)	-0.76	-0.76	0.70	1	0.77	-0.71
CCD (mm)	-0.68	-0.58	0.65	0.76	1	-0.62
TA length (mm)	0.75	0.74	-0.41	-0.71	-0.62	1

FBM= foot bimalleolar angle; MMN= medial malleolar navicular distance; MST= medial soft tissue thickness; CCA= calcaneocuboid angle; CCD= calcaneocuboid distance; TA= tendon-achilles length

## Discussion

CTEV is one of the common congenital foot anomalies^1-5^. Dyer *et al* stated that Pirani score was very reliable, easy to memorise and was better than Dimeglio score, so Pirani score was taken to grade severity in our study^13^.

FBM angle uses the foot print to quantify the CTEV deformity depending on the objective assessment of calcaneal rotation. FBM angle is a combined indirect indicator of forefoot adduction and the hind foot varus (which are the main variants of the club foot deformity), although it does not give the access to the component of hindfoot deformities by the Pirani score^11^. Jain *et al* found 82.5 degrees to be the normal FBM angle in their study^10^. It is a cost-effective method as it only requires a sheet of paper and inkpad^11^.

Ultrasound was found to be a simple and reproducible investigation. The average time taken was about 15-20 minutes per foot and examination was done when the baby was asleep. The medial view was used to assess medial malleolus, navicular and talus. It clearly demonstrated the position of the displaced navicular and measured the medial malleolar-navicular (MMN) distance and medial soft tissue thickness (MST). With serial casting the MMN distance showed progressive increase and MST was found to decrease along the course of treatment. When these parameters (MMN and MST) were correlated it was found that there was negative correlation between them which was in concurrence with Khaled *et al* who also found negative correlation between them in their study^9^ (Table II).

**Table II:** Mean medial malleolar navicular (MMN) distance and mean medial soft tissue thickness (MST) values

		At the initiation of treatment	At 4th week	At the end of treatment
MMN (mm)	Mean	0.32	0.43	0.68
	SD	0.09	0.12	0.10
MST (mm)	Mean	1.25	1.19	1.06
	SD	0.06	0.10	0.09

MMN= medial malleolar navicular distance; MST= medial soft tissue thickness

On the lateral view the calcaneocuboid relationship was assessed. Calcaneocuboid angle is an indicator of medial deviation of cuboid which is essential as, if left untreated, it results in residual deformity. Calcaneocuboid distance (CCD) was also measured. With serial casting both the CCA and CCD showed progressive decrease in the course of treatment. CCA and CCD showed positive correlation in this study which was similar to observation made by Khaled *et al*^9^ (Table III).

**Table III:** Mean calcaneocuboid angle (CCA) and mean calcenocuboid distance (CCD) values

		At the initiation of treatment	At 4th week	At the end of treatment
CCA (degree)	Mean	21.30^o^	18.63^o^	23.71^o^
	SD	2.24	5.77	10.24
CCD (mm)	Mean	0.23	0.18	0.08
	SD	0.06	0.06	0.05

CCA= calcaneocuboid angle; CCD= calcaneocuboid distance

On the posterior view, the length of tendo-achilles was found to be (mean 2.99, SD 0.36) at first visit, (mean 3.17, SD 0.41) at second visit and (mean 3.91, SD 0.29) at completion of treatment in this study. On posterior projection, with serial casting, it was found that length of Achilles tendon showed progressive increase in the course of treatment. Generally^5-6^ casts were required for the complete correction of foot, with requirement of tenotomy in some.

Limitations of the study were firstly that there can be intraobserver and interobserver variation in calculating FBM angle, and secondly the duration of this study and number of patients were not sufficient to predict long term results.

## Conclusion

Our study showed positive correlation between FBM angle and ultrasound parameters during treatment of CTEV. Ultrasound has the potential to accurately depict the patho-anatomy in clubfoot. FBM angle and ultrasound are objective methods to assess the severity of clubfoot. FBM angle and ultrasonography correlated in severity of deformity and correction achieved in the course of treatment.

## Conflict of Interest

The authors declare no conflicts of interest.
